# Oral Dysbiosis in Severe Forms of Periodontitis Is Associated With Gut Dysbiosis and Correlated With Salivary Inflammatory Mediators: A Preliminary Study

**DOI:** 10.3389/froh.2021.722495

**Published:** 2021-10-11

**Authors:** Dione Kawamoto, Rodrigo Borges, Rodolfo Alvarenga Ribeiro, Robson Franciso de Souza, Pâmela Pontes Penas Amado, Luciana Saraiva, Ana Carolina Ratto Tempestini Horliana, Marcelo Faveri, Marcia Pinto Alves Mayer

**Affiliations:** ^1^Department of Microbiology, Institute of Biomedical Sciences, University of São Paulo, São Paulo, Brazil; ^2^Laboratório de Biologia Computacional e Bioinformática, Centro Internacional de Pesquisa (CIPE) - A.C. Camargo Cancer Center, São Paulo, Brazil; ^3^Division of Periodontology, Department of Stomatology, School of Dentistry, University of São Paulo, São Paulo, Brazil; ^4^Biophotonics Applied to Health Sciences, University Nove de Julho, São Paulo, Brazil; ^5^Dental Research Division, Department of Periodontology, Guarulhos University, Guarulhos, Brazil

**Keywords:** periodontitis, oral microbiome, fecal microbiome, 16SrRNA sequencing, dysbiosis

## Abstract

Inflammation is a driven force in modulating microbial communities, but little is known about the interplay between colonizing microorganisms and the immune response in periodontitis. Since local and systemic inflammation may play a whole role in disease, we aimed to evaluate the oral and fecal microbiome of patients with periodontitis and to correlate the oral microbiome data with levels of inflammatory mediator in saliva.

**Methods:** Nine patients with periodontitis (P) in Stage 3/Grade B and nine age-matched non-affected controls (H) were evaluated. Microbial communities of oral biofilms (the supra and subgingival from affected and non-affected sites) and feces were determined by sequencing analysis of the *16SrRNA* V3–V4 region. Salivary levels of 40 chemokines and cytokines were correlated with oral microbiome data.

**Results:** Supragingival microbial communities of P differed from H (Pielou's evenness index, and Beta diversity, and weighted UniFrac), since relative abundance (RA) of *Defluviitaleaceae, Desulfobulbaceae, Mycoplasmataceae, Peptostreococcales-Tissierellales, and Campylobacteraceae* was higher in P, whereas *Muribaculaceae* and *Streptococcaceae* were more abundant in H. Subgingival non-affected sites of P did not differ from H, except for a lower abundance of *Gemellaceae*. The microbiome of affected periodontitis sites (PD ≥ 4 mm) clustered apart from the subgingival sites of H. Oral pathobionts was more abundant in sub and supragingival biofilms of P than H. Fecal samples of P were enriched with *Acidaminococcus, Clostridium, Lactobacillus, Bifidobacterium, Megasphaera, and Romboutsia* when compared to H. The salivary levels of interleukin 6 (IL-6) and inflammatory chemokines were positively correlated with the RA of several recognized and putative pathobionts, whereas the RA of beneficial species, such as *Rothia aeria* and *Haemophilus parainfluenzae* was negatively correlated with the levels of Chemokine C-C motif Ligand 2 (CCL2), which is considered protective. Dysbiosis in patients with periodontitis was not restricted to periodontal pockets but was also seen in the supragingival and subgingival non-affected sites and feces. Subgingival dysbiosis revealed microbial signatures characteristic of different immune profiles, suggesting a role for candidate pathogens and beneficial organisms in the inflammatory process of periodontitis.

## Introduction

The dysbiotic microbiota in periodontitis-affected subgingival sites is characterized by an increased abundance of pathogens and pathobionts whereas the abundance of genera considered as beneficial to the host is decreased [1, 2]. Animal experimental studies suggested that the periodontal pathogen, *Porphyromonas gingivalis* might induce dysbiosis not only in the oral cavity, but also in the gut, which affects the integrity of the gut epithelial barrier, and consequently increases systemic inflammation [3, 4]. Furthermore, dysbiosis found not only in the oral cavity, but also in the gut is a frequent finding in most of the conditions associated with periodontitis, such as arthritis [5], obesity [6, 7], diabetes [8], and inflammatory bowel disease [9]. However, to date, most studies on the microbiome of periodontitis have focused on the comparison between microbial communities of subgingival biofilms from periodontal pockets and those from healthy subjects [1, 2, 10] with few exceptions [11, 12].

There is also evidence of altered gut microbiome in Grade B periodontitis (previously known as chronic periodontitis) [12], and Grade C periodontitis of the molar incisor pattern (previously known as localized aggressive periodontitis) [13]. These observations led to the hypothesis that alterations in the gut microbiome play a key role in periodontitis and its association with inflammatory diseases [14–17].

When the balance between the host and the subgingival microbiome is disrupted, pathogens and pathobionts trigger host-defense mechanisms, leading to inflammation and bone resorption [18]. Inflammation is a driven force to modify the microbial community resulting in a continuous cycle of dysbiosis, an immune response, and tissue breakdown [19, 20]. The environmental conditions of inflamed periodontal pockets, such as low oxygen levels, enriched nutrition derived from the breakdown of host proteins, and high gingival fluid volume, together with synergistic microbial interactions, favor inflammophilic, anaerobic, proteolytic, and fastidious organisms. Gingival inflammation also influences the microbial composition of supragingival plaque [21], and the microbial composition of subgingival sites is profoundly affected by the supragingival dental plaque [22].

Thus, unresolved exacerbated inflammation characteristic of chronic periodontitis is associated with high levels of inflammatory mediators in the gingival tissues [23, 24], crevicular fluid [25], and saliva [26, 27]. However, little is known about the contribution of the microbial community to the pattern of the inflammatory mediator seen in subjects with periodontitis, with few exceptions [28], as most data rely on the role of selected pathogens [29].

Thus, we evaluated the microbiome of oral dental plaque and feces of patients in Stage III, Grade B periodontitis (previously known as chronic periodontitis) and compared these data with those of age-matched periodontally healthy subjects. In addition, we evaluated the salivary levels of inflammatory mediators and correlated them with the oral microbiome.

## Methods

### Study Design and Groups

This study was conducted according to the Declaration of Helsinki of 1975 on experimentation involving human subjects and approved by the Research Ethics Committee of the Biomedical Sciences Institute of University of São Paulo (CAAE 42056614.3.0000.5467) and associated institutions. Subjects were informed about the study objectives and signed an “Informed and Free Consent Form.” Patients, aged between 35 and 55 years and healthy age-matched, were selected at the School of Dentistry of University of São Paulo (São Paulo, SP, Brazil), the Periodontal Clinic of Guarulhos University (Guarulhos, SP, Brazil), and the School of Dentistry of Nove de Julho University (São Paulo, SP, Brazil).

### Clinical Assessments

Clinical measurements were performed by calibrated periodontists. The clinical parameters evaluated were bleeding on probing (BoP) (no = zero/ yes = 1), probing depth (PD), and clinical attachment level/loss (CAL), measured at six sites per tooth in all teeth (excluding third molars), using a periodontal probe (Hu-Friedy^®^, Chicago, IL, USA).

### Eligibility Criteria

Subjects (*n* = 9) with periodontitis Stage III and a moderate rate of progression (Grade B) (P) [30, 31] comprised patients aged between 35 and 55 years, with at least 20 teeth, more than 30% of sites with CAL and PD >3mm, at least one site with CAL ≥5mm and radiographic bone loss extending at least to the middle third of the root, percentage of bone loss/age ranging from 0.25 to 1.0. The control group (H) consisted of periodontally healthy subjects (*n* = 9) without sites with PD and CAL measurements >3 mm, <20% of sites exhibiting BoP and no extensive caries of lesions and at least 28 permanent teeth [32]. Exclusion criteria included pregnancy, smoking, current or previous periodontal treatment, presence of systemic diseases, use of medications that could affect the periodontium or immune response, and use of systemic antibiotics and/or mouthwashes containing antimicrobials in the previous 3 months. All subjects diagnosed with periodontitis received the required periodontal treatment after sample collection.

### Microbiome Sample Collection

Biofilm samples of P subjects were collected as follows: the supragingival biofilm was obtained from the buccal or the lingual non-affected sites (PD = 0–3 mm); the subgingival samples of non-affected (PD = 0–3 mm) and affected sites (PD > 4 mm) were obtained at the interproximal sites, after the removal of the supragingival biofilm. Supragingival and subgingival biofilm samples were collected from healthy individuals from randomized sites. Samples collected using Gracey mini-five curettes (Hu-Friedy^®^, Chicago, IL, USA) were obtained from four teeth at each location (the supra or the subgingival) and condition (affected or non-affected sites) from each individual and pooled according to the site and location in Tris -EDTA buffer (TE) (10 mM Tris-HCl, 0.1 mM ethylenediaminetetraacetic acid, pH7.6). Fecal samples were self-collected using a sterilized recipient. Individuals were asked to store the specimen at −20°C and transported it in a styrofoam box with recyclable ice. All samples were stored at −80°C until manipulation.

### DNA Extraction and 16SrRNA Gene Sequencing

Total genomic DNA of oral biofilms was extracted using Meta-G-Nome™ DNA Isolation Kit (Epicentre Biotechnologies, Madison, WI, USA) according to the protocol of the manufacturer. Stool DNA was extracted using the QIAamp^®^ DNA Stool Mini Kit (Qiagen, Hilden, Germany). DNA quality and amount were determined using Qubit dsDNA HS Assay Qubit Fluorimeter 2.0 (ThermoFisher Scientific, Carlsbad, CA, USA).

The hypervariable V3–V4 region of *16SrRNA* was amplified using the primers, Bakt_341F CCTACGGGNGGCWGCAG and Bakt_805R GACTACHVGGGTATCTAATCC [33]. Amplicons were sequenced by Macrogen (Seoul, Republic of Korea) by high-throughput sequencing using Illumina MiSeq 2 × 250 platforms (Illumina Inc., CA, USA). The sequence data are available at https://www.ncbi.nlm.nih.gov/bioproject/735261.

### Sequencing Data Processing and Statistical Analyses

Sequencing data were analyzed using Quantitative Insights into Microbial Ecology (QIIME2) 2020.6 [34]. The demultiplexed sequences were merged and the sequences were trimmed in the region flanked by sequencing primers, Bakt_341F and Bakt_805R [35]. Sequencing reads were filtered for the length of 230 bp and with a minimum overlap of 8 bp and analyzed using the DADA2 software package [36]. Checking, filtering for chimera, and clustering were performed using VSEARCH (https://github.com/torognes/vsearch).

Alpha-diversity indices, such as Faith's phylogenetic diversity (community richness) and Pielou's evenness (community evenness) were calculated.

Beta-diversity group analysis was performed using a Weighted UniFrac matrix [37], and divergence between the groups was highlighted by Principal Coordinates Analysis (PCoA). Differences between groups of samples of Periodontitis and H were estimated by the analysis of similarity using UniFrac. Taxonomy was assigned to each amplicon sequence variant (ASV) based on Silva 138 database [38]. Oral ASVs were then identified by using HOMD 15.1 database [39].

Oral and fecal core microbiomes were estimated with ASVs present in at least 70% of the samples and the Venn diagram was applied [40].

### Saliva Collection and Cytokine and Chemokine Analysis

Unstimulated whole saliva samples were obtained. The levels of chemokines and cytokines in the saliva samples were evaluated by a Bio-Plex Pro™ Human Chemokine assay kit (Bio-Rad, Hercules, CA, USA) following the instructions of the manufacturer, as described in http://www.bio-rad.com/webroot/web/pdf/lsr/literature/Bulletin_6499.pdf. Detailed information on these procedures was previously described [27].

### Statistical Analyses

Sample calculation for microbiome and inflammatory mediators was based on data from a pilot study using four samples from each group. The relative abundance (RA) at the phylum level was taken as an endpoint, considering 0.37 ± 0.23 (mean ± SD) for the P group and 0.49 ± 0.41 for the H group. The pro-inflammatory/anti-inflammatory ratio was taken as the endpoint, considering 1.45 ± 0.7 (P group) and 0.78 ± 0.2 (H group). Considering a power of 80% and a significance level of 5%, a minimum of nine individuals per group would be required. Analysis was performed using the BioEstat^®^ software V5.3.

Wilcoxon-Mann-Whitney test was performed to detect the differences in alpha, diversity, clinical parameters, and differences in RA between groups, considering the statistical difference when *p* < 0.05.

Weighted UniFrac similarity matrices were calculated to compute the similarities between the groups, and the distances were compared using Permutational Multivariate Analysis of Variance (PERMANOVA) in Qiime2. After testing the distribution by Shapiro-Wilk normality tests, a binomial test was applied to analyze the inter-group differences. Correlation between the RAs of the species in the subgingival samples pooled according to the site condition, in order to evaluate the differences between the affected and non-affected sites, cytokines, and the salivary levels of chemokines was calculated using Spearman's rank coefficient, considering a significance level of *p* < 0.05. For these analyses, R Studio 3.6 Software with packages Rstatix, Survive, ggplot2 and dplyr, corrplot, and Hmisc was used.

## Results

### Clinical Characteristics

Eighteen subjects aged 35–55 years, who had never been submitted to periodontal treatment, formed the studied population. As expected, periodontal clinical parameters differed significantly between periodontitis and the health group (Wilcoxon-Mann-Whitney test, *p* < 0.05), as shown in Table 1.

**Table 1:** Clinical characteristics of the study population.

**Condition**		**Periodontitis (*n* = 9)**	**Health (*n* = 9)**
Age (years)		43.5 (±5.88)	40.62 (±3.37)
Gender (%)	Male	77.77	66.66
	Female	22.22	33.33
BoP (mm)		54.88 (±26.49)**	12.05 (±10.15)
CAL (mm)		4.44 (±0.79)***	2.01 (±0.66)
PD (mm)		4.18 (±0.59)***	2.02 (±0.66)

Statistical difference was considered when p < 0.05. For results with p < 0.01** and for p < 0.001*** by Wilcoxon-Mann-Whitney test.

### Sequence Profile Analysis of the Oral and Feces Microbiome

Sixty-one samples were evaluated, 45 pooled oral biofilms (nine from the supragingival biofilm from each group, nine from the subgingival non-affected sites, nine from the affected sites of P, and nine from the subgingival sites of (H) and 16 fecal samples, which demultiplexed the sequences and generated 7,108,527 paired-end reads. Two fecal samples (one from each group) were lost due to a lack of collaboration from the subjects.

After filtering to the specific region of sequence primers, Bakt_341F and Bakt_805R and removing the denoised reads, 2,340,642 paired-end reads were generated, with an average of 38,371.18 reads per sample (min 15,177 and maximum 64,435 reads). The average of reads per sample was 39,832.02 in oral and 34,262.56 in fecal samples. Rarefaction analysis determined that 14,000 reads were needed for sampling depth, according to the number of observed ASVs and number of samples (Supplementary Figure 1).

A total of 691 ASVs were detected, distributed among 17 phyla, 32 classes, 81 orders, 108 families, and 234 genera in fecal and oral samples, and classified using the SILVA138 database. With the HOMD15 database, 519 ASVs were distributed at the phylum level (12), class (23), order (39), genus level (118), and species (410). The remaining 172 ASVs were not classified by HOMD15.

### Dysbiosis in the Supragingival Microbiome of Periodontitis

**Figure 1:**
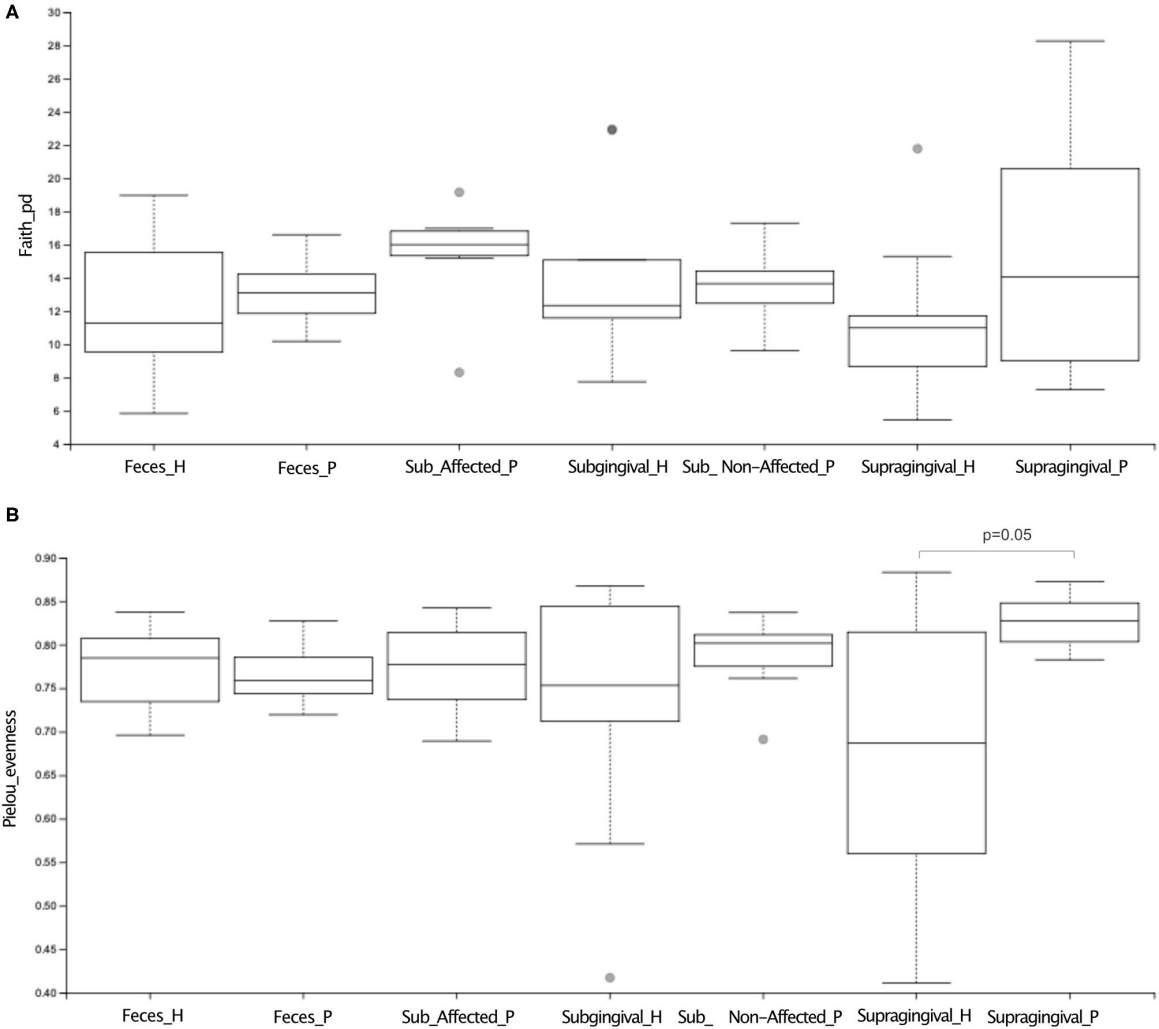
Alpha diversity analysis of microbiome of oral (supragingival, subgingival non-affected sites; subgingival affected sites) and fecal samples of periodontitis (P) and health (H) subjects. In **(A)** richness (Faith's PD) index and **(B)** evenness (Pielou) index analysis. Differences were considered significant when *p* ≤ 0.05 using Wilcoxon-Mann Whitney test.

Alpha diversity indices of richness [Faith's PD)] revealed no differences in the supragingival microbial communities between P and H (Figure 1A, Supplementary Table 1). However, the supragingival microbiome of P showed a higher Pielou's index (evenness) than that of H (Figure 1B). (Wilcoxon-Mann-Whitney test, *p* ≤ 0.05).

**Figure 2:**
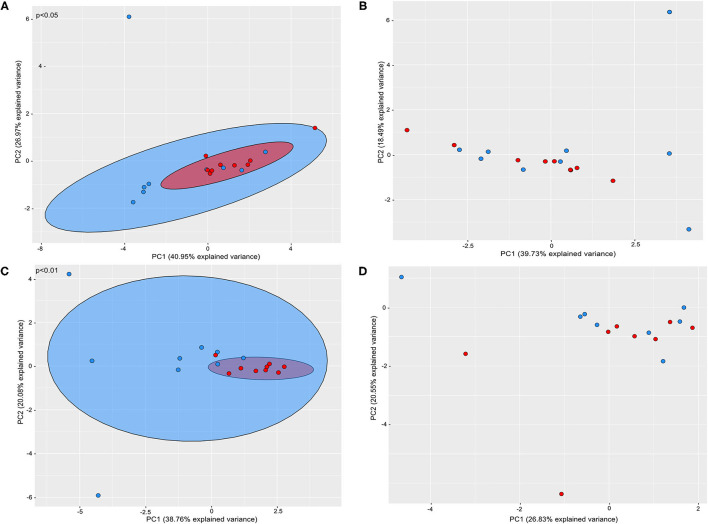
Principal coordinate analysis based on weighted UniFrac distance metric. Graphics represents beta diversity analysis between samples of P and H groups: **(A)** supragingival sites; **(B)** subgingival non-affected sites; **(C)** subgingival affected sites of P and subgingival sites of H **(D)** feces of H and P. Red dots correspond to samples of Periodontitis patients and blue dots to samples of health subjects. A significance level of 5% was applied by using PERMANOVA test.

Beta diversity analysis revealed that the supragingival microbiomes of periodontitis subjects clustered apart from those of healthy subjects [Weighted UniFrac, PERMANOVA test, *p* ≤ 0.05] (Figure 2A).

**Figure 3:**
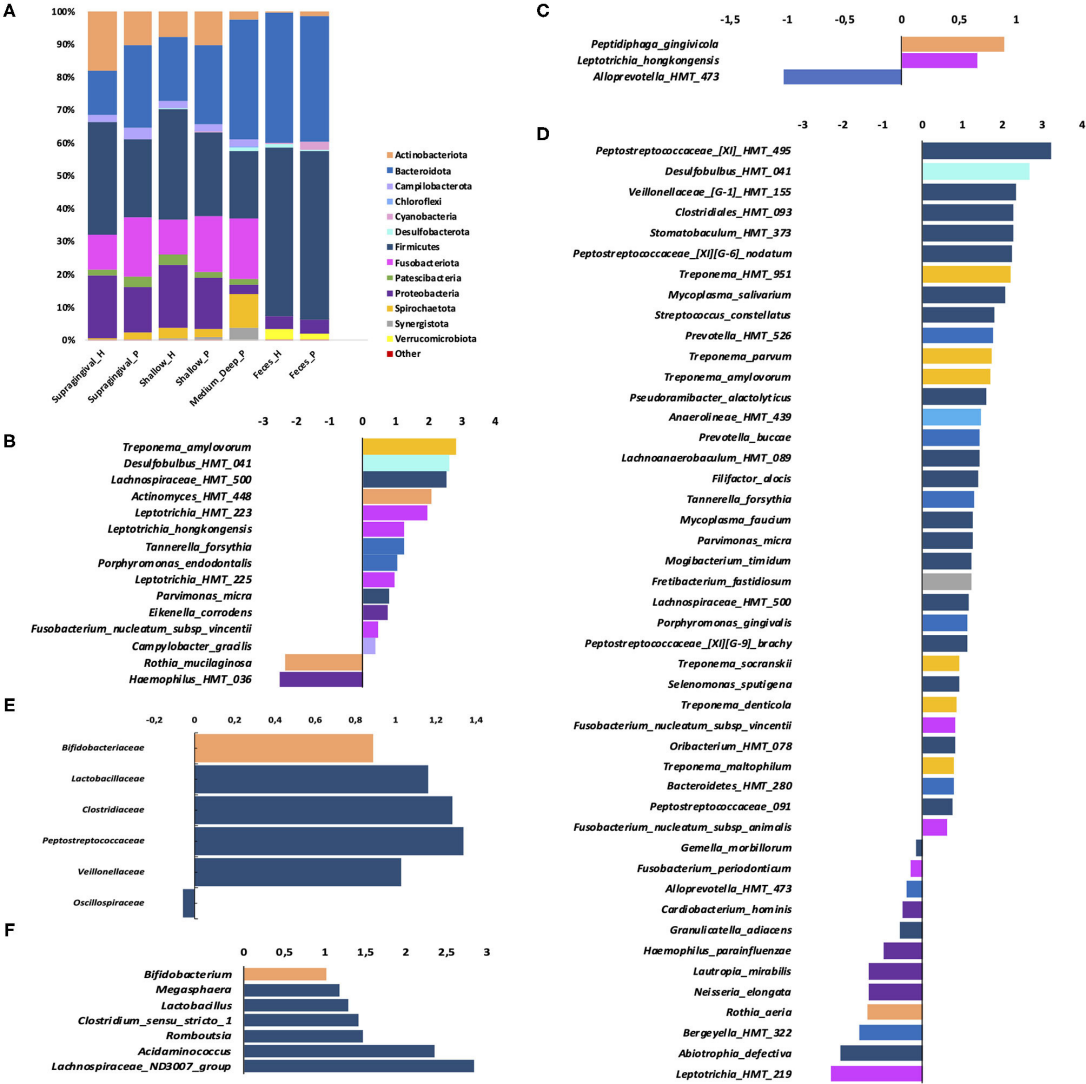
Phylum distribution in samples of the oral sites and feces **(A)**; **(B–D)**: Fold changes (log_2_) relative abundance of species in periodontitis samples (positive values) compared to control (negative values)—in: **(B)** supragingival biofilm **(C)** subgingival non- affected sites of periodontitis patients and health patients; **(D)** subgingival affected sites of periodontitis patients compared to subgingival sites of H; **(E)** fold changes of Relative abundance of families in feces and **(F)** genera in feces. Only species which the relative abundance differed between P and H **(B–D)** are shown (Wilcoxon-Mann Whitney test, *p* ≤ 0.05).

At the phylum level, Firmicutes were more abundant in H, whereas Bacteroidota and Campilobacterota were more abundant in P than H (Figure 3A).

The families *Defluviitaleaceae, Desulfobulbaceae, Mycoplasmataceae, Peptostreococcales-Tissierellales, and Campylobacteraceae* were more abundant in P, whereas *Muribaculaceae* and *Streptococcaceae* were more abundant in H (Supplementary Figure 2A). At the genus level, *Porphyromonas, Fusobacterium, Parvimonas, Campylobacter, Mycoplasma, Desulfobulbus, Oribacterium, Veillonella*, and *Defluviitaleaceae* UCG-011 were more abundant in P than H, whereas *Streptococcus* and *Actinobacillus* were more abundant in H (Supplementary Figure 2B). The ASVs classified at the species level, which differed in abundance between P and H, are shown in Figure 3B.

### Dysbiosis in the Subgingival Biofilm of Periodontitis

Alpha (Figure 1, Supplementary Table 1) and Beta diversities analysis did not reveal differences between the subgingival microbial communities of non-affected sites of P and H (Figure 2B). The RA of different bacterial groups at these sites did not differ at the phylum and class levels. However, the subgingival sites of H revealed a higher abundance of *Gemellaceae* than the non-affected sites of P [median (interquartile range) = 0.02 (0.02–0.04) in H vs. 0.008 (0.005–0.01) in P]. The genera *Parvimonas, Atopobium*, and *Fusobacterium* were more abundant in the subgingival non-affected sites of P than H, whereas *Actinobacillu*s was more abundant in health (Supplementary Figure 2C). There were also differences in the RA between the groups at the species level (Figure 3C).

Alpha diversity indices of richness and evenness did not differ when the microbiome of the affected sites of P was compared to the subgingival sites of H (Figure 1, Supplementary Table 1). However, Beta diversity analysis indicated that the subgingival samples from the affected sites of P differed from the subgingival samples of periodontally healthy subjects (H) (Weighted UniFrac, *p* < 0.01, PERMANOVA test), as shown in Figure 2C. The RA of bacterial groups of the affected sites of P and the subgingival sites of H differed in all the taxonomic levels. The phyla, Bacteroidota, Desulfobacterota, Fusobacteriota, Spirochaetota, Synergistota, and Chloroflexi were more abundant in the affected sites of P, whereas Actinobacteriota and Firmicutes were more abundant in H (Wilcoxon-Mann-Whitney test, *p* < 0.05) (Figure 3A). Differences in RA between the two groups at the family level are described in Supplementary Figure 2D. The genera, *Atopobium, Porphyromonas, Prevotella, Tannerella, Flexilinea, Desulfobulbus, Mycoplasma, Pseudoramibacter, Oribacterium, Stomatobaculum, Mogibacterium, Filifactor, Parvimonas, Fusobacterium, Streptococcus, Treponema*, and *Fretibacterium* were more abundant in the diseased sites of P while *Actinomyces, Rothia, Bergeyella, Abiotrophia, Granulicatella, Gemella, Lautropia, Neisseria, Actinobacillus*, and *Haemophilus* were more abundant in H, as shown in Supplementary Figure 2E. The RA of several species differed between the subgingival affected sites of P and the subgingival sites of H (Figure 3D).

### Correlation Between Cytokines and Chemokines in Saliva and the Oral Microbiome

Data of the mean levels of cytokines and chemokines (pg/ml) are described in Supplementary Table 2. Abundances of species at the subgingival affected sites of P and subgingival sites of H were correlated with the levels of salivary inflammatory mediators (as shown in Figure 3D and detailed in Supplementary Table 2). Furthermore, only the species detected in seven out of nine patients of each group were evaluated (binomial test, *p* > 0.4).

**Figure 4:**
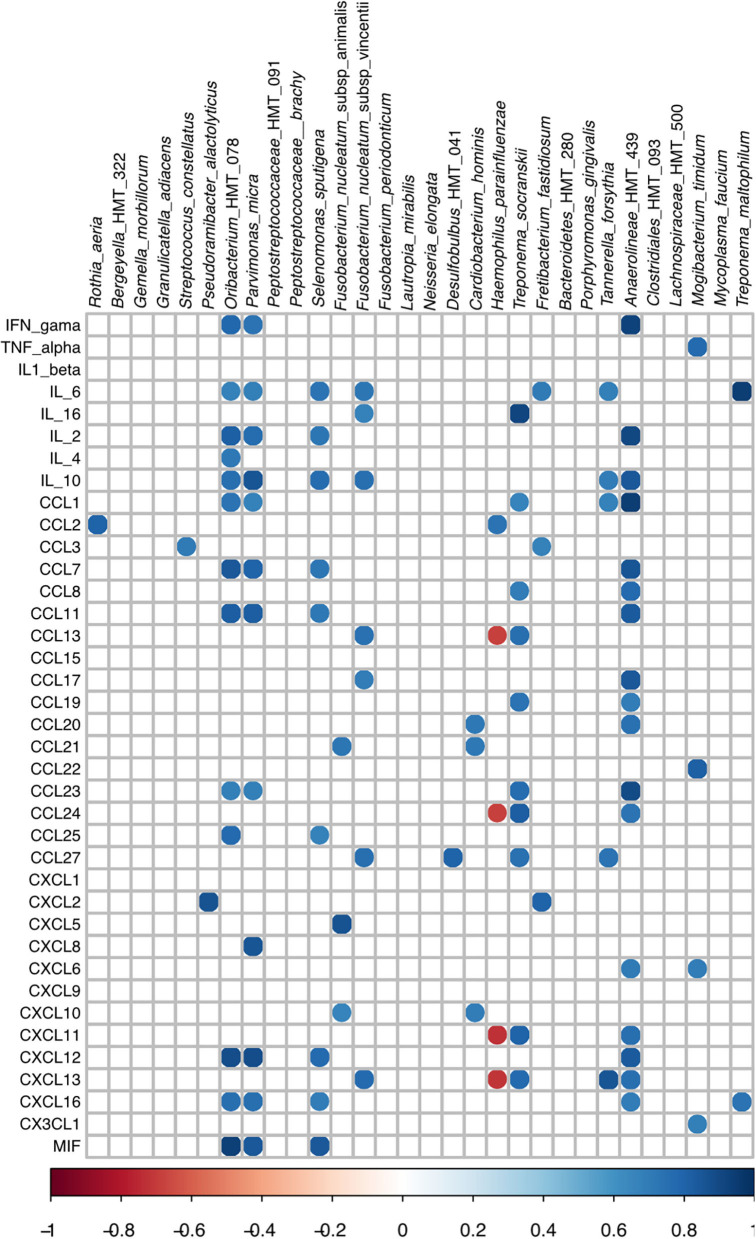
Spearman *Rho* correlation among RA values of subgingival bacteria and cytokines/chemokines levels in saliva of periodontitis subjects. Only species more or less abundant in subgingival affected sites of periodontitis than in subgingival sites of the healthy patients were evaluated.

Data on positive and negative correlations between RA of AVS in the subgingival affected sites and the salivary levels of inflammatory mediators of the Periodontitis group are shown in Figure 4. Only correlations with Rho values >0.67 or <-0.67 were considered when *p* ≤ 0.05.

Other positive and negative correlations were seen in healthy subjects when the RA of AVS of the subgingival sites differing between H and P and the inflammatory levels of mediators were evaluated (Supplementary Figure 4).

### Altered Gut Microbiome of Patients With Periodontitis

Alpha (Figure 1, Supplementary Table 1) and beta diversities indices revealed no differences between the fecal microbial communities of H and P (Figure 2). There were no differences in the abundance of different Phyla and Orders in the fecal samples between P and H. Nevertheless, the classes, *Bacteroidia* and *Actinobacteria* were more abundant in H than in P [Median (interquartile range)]: [*Bacteroidia* 1.49 (1.41–1.68) in H and 1.17 (1.03–1.27) in P, *p* ≤ 0.05] [*Actinobacteria* 0.002 (0–0.004) in H and 0.02 (0.012–0.03) in P, *p* ≤ 0.05]. Furthermore, the fecal samples from P were enriched in several families and the genera of Firmicutes when compared to H, as shown in Figures 3E,F, respectively.

### Oral and Fecal Core Microbiomes

**Figure 5:**
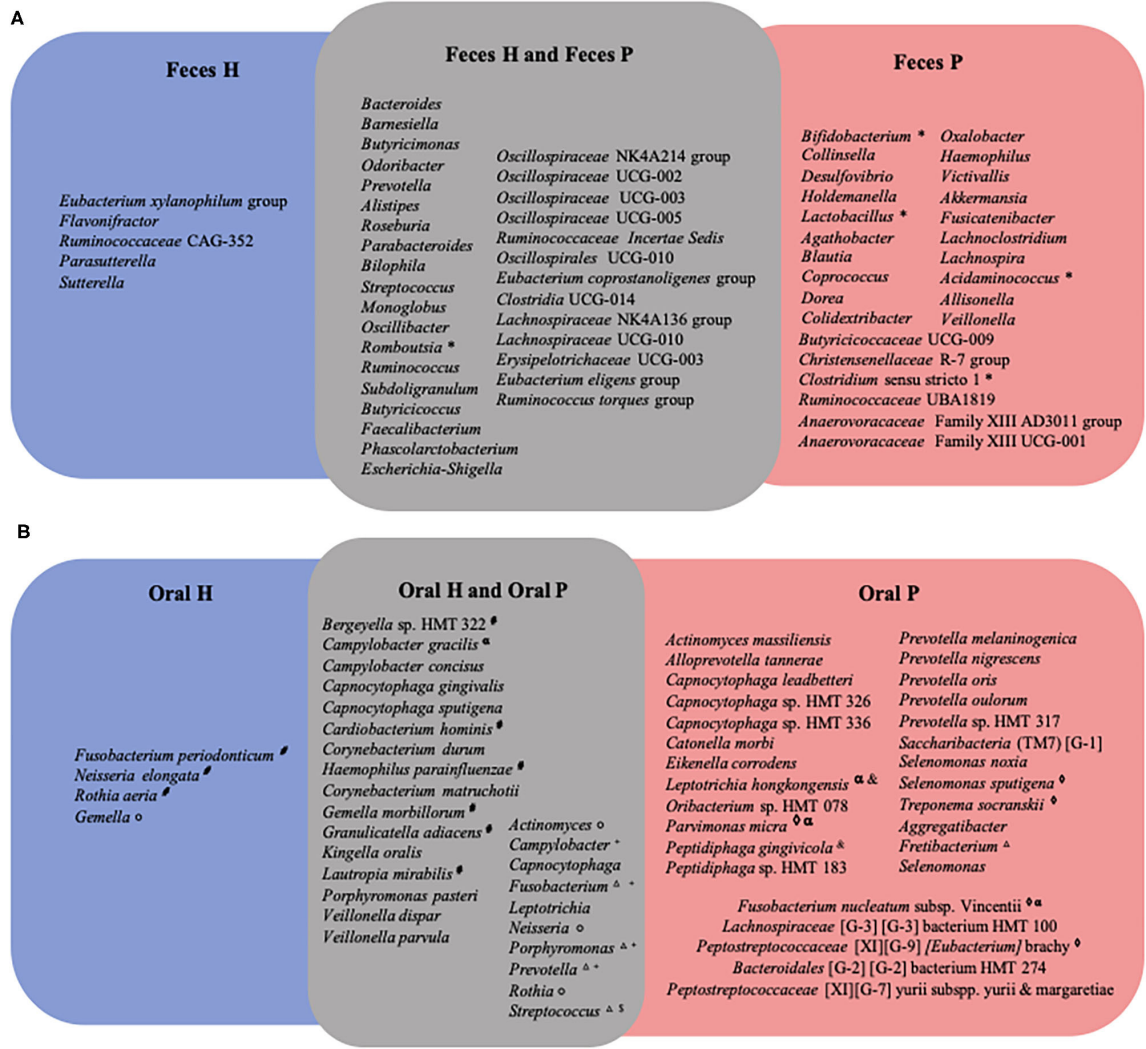
Venn diagram of core microbiome representing bacteria genera or specie present in at least 70% of subjects. In **(A)** genera in feces of periodontitis patients (Feces_P) and periodontally healthy individuals (Feces_H); in **(B)** species in oral biofilm of periodontitis patients (Oral_P) and oral biofilm periodontally healthy individuals (Oral_H). For (*) genera that were more abundant in feces of periodontitis patients in comparison of feces of H; (^
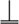
^) genera more abundant in P when subgingival affected sites of P group were compared to shallow sites of H; (°) genera more abundant in H when subgingival affected sites of P group were compared to subgingival sites of H; (^+^) genera more abundant in P when supragingival sites of P were compared to supragingival sites of H; (^$^) genera more abundant in H when supragingival sites of P were compared to supragingival sites of H; (^Δ^) genera more abundant in P when subgingival shallow sites of P were compared to subgingival shallow sites H; (^α^) species more abundant in supragingival sites of P than supragingival sites of H; (^&^) species more abundant in subgingival non-affected sites of P than subgingival sites of H; (^♢^) species more abundant in subgingival affected sites of P group when were compared to subgingival sites H; (^#^) species more abundant in subgingival sites of H when compared to subgingival affected sites of P group.

Core microbiome analysis showed differences in the distribution of several genera in feces (Figure 5A), and on the distribution of several genus and species in the oral cavity when patients with periodontitis and health individuals were compared (Figure 5B). Abundances of oral and gut bacteria in both groups were correlated (Supplementary Figure 5). Moreover, site-specificity was accessed by comparing the oral and fecal microbiome of P and H (Supplementary Figure 3). *Streptococcus* and *Prevotella* were found both at the oral cavity and feces of H and P (Supplementary Figures 3A,B), whereas *Veillonella* and *Haemophilus* were common to both sites only in the P group (Supplementary Figure 3A), and *Clostridia* UCG14 was detected at the oral cavity and feces of H (Supplementary Figure 3B).

## Discussion

Thus, we aimed to evaluate the microbial communities of non-treated patients with periodontitis by accessing the microbiomes of supra and subgingival sites, and feces and to correlate the oral microbiome with levels of inflammatory mediators in saliva.

The studied population comprised periodontitis subjects who were compared to age-matched periodontal healthy subjects, in consonance with other studies [2, 41, 42]. Only grade B patients with periodontitis (moderate rate of progression) were selected and age was limited at 55 years. These approaches were relevant due to the increased inflammation with the aging process, which may compromise the evaluation of inflammatory mediators, and their influence on the resident microbial communities [43].

Our data revealed that the microbiome of periodontal pockets clustered apart from that of the gingival crevice of health subjects (Figure 2C). The health-associated subgingival microbiome was characterized by a higher abundance of Actinobacteriota and Firmicutes, whereas periodontitis sites harbored a higher abundance of Bacteroidota, Desulfobacterota, Fusobacteriota, Spirochaeota, Synergistota, and Chloroflexi. Richness and evenness (Pielou) diversity indices did not differ between the samples of the periodontal pockets and the subgingival sites of H, as previously reported [11, 44]. These data are in contrast to studies that indicated a lower [45, 46], or a higher diversity and richness [1, 2, 10] in disease than in health, possibly due to differences in sampling methods and/or disease severity. Previous studies described higher abundances of the phyla, Spirochaeota, Synergistota, Bacteroidota, and Fusobacteriota in disease [10, 44, 47], although this is still not a consensus [1, 46]. Our data confirmed the association of periodontitis with increased levels of pathobionts of the genera, *Atopobium, Porphyromonas, Prevotella, Tannerella, Flexilinea, Desulfobulbus, Mycoplasma, Pseudoramibacter, Oribacterium, Stomatobaculum, Mogibacterium, Filifactor, Parvimonas, Fusobacterium, Treponema*, and *Fretibacterium* and the decreased abundance of *Actinomyces, Rothia, Bergeyella, Abiotrophia, Granulicatella, Gemella, Lautropia, Neisseria, Actinobacillus*, and *Haemophilus* when compared to health. The association of most of these genera with disease or periodontal health has been previously shown [10, 11, 41, 45–50]. Although most of the organisms which abundantly increased in health were previously considered beneficial, the data on *Gemella morbillorum* are conflicting [10, 12, 51, 52].

Not surprisingly, the microbial composition of the supragingival biofilms also differed between periodontitis and health groups, differing from data reported by Galimanas et al. [11]. Supragingival plaque of diseased subjects was enriched by recognized pathobionts of the genera, *Porphyromonas, Fusobacterium, Parvimonas, Mycoplasma, Desulfobulbus, Oribacterium*, and *Campylobacter*, but also by *Veillonella*, which was not previously related to the disease and not yet characterized by Defluviitaleaceae UCG-011. On the other hand, the supragingival biofilm of health subjects exhibited a higher abundance of *Streptococcus* and *Actinobacillus* than the supragingival biofilm of P subjects. Several ASVs are more abundant in the supragingival biofilm of periodontitis subjects, such as *Tannerella forsythia, Fusobacterium nucleatum* subsp. *vincentii, Porphyromonas endodontalis, Campylobacter gracilis, Eikenella corrodens, Leptotrichia hongkongensis, Desulfobulbus* HMT 041, and *Treponema amylovorum* were previously associated with periodontal pockets [1, 10, 42, 47, 52–54].

Early studies using target microbial techniques indicated that the supragingival biofilm can be a source of pathobionts [55, 56], whose growth would possibly be supported by inflammatory conditions in the nearby gingival tissues [57]. Thus, our data extend the repertoire of organisms considered as biomarkers of supragingival plaque in periodontitis [11], and include organisms, such as *Veillonella*, with no pathogenic potential, but which may find the suitable conditions for growth in the supragingival plaque of P [56]. Nevertheless, further studies should demonstrate whether regular supragingival plaque control and subgingival mechanical treatment can reestablish the supragingival microbiome compatible with health.

The microbial compositions of non-affected subgingival sites of P and healthy subjects were similar. Indeed, only the family, *Gemellaceae* and the genus, *Actinobacillus* were more abundant in the subgingival sites of H than in the non-affected sites of P, whereas known pathobionts, such as *Parvimonas, Atopobium*, and *Fusobacterium* were more abundant in the subgingival non-affected sites of P. Thus, our data suggested that the subgingival non-affected sites in patients with periodontitis, even those without signs of inflammation (no BoP) could be a transitory ecosystem to the disease, since deeper pockets of diseased subjects act as reservoirs for the spread of infection to healthy sites, as hypothesized in the early studies [58].

The association of *Rothia, Haemophilus, Neisseria, Streptococcus, Actinobacillus, Gemella, Abiotrophia, Lautropia*, and *Granulicatella* with health, as reported previously [10, 41, 45, 48, 49], was reinforced by their decreased abundance, not only in periodontal pockets but also in the supra and subgingival healthy sites of the P group.

Oral dysbiosis in patients with periodontitis was followed by an altered gut microbiome, despite the absence of other diseases in this group. Our data are in accordance with a study that reported no differences in diversity in the fecal microbiome of periodontitis, gingivitis, and H subjects [12]. However, the classes, *Bacteroidia* and *Actinobacteria* were more abundant in the fecal samples of H than in P. On the other hand, the fecal samples of P were enriched with several Firmicutes, including the families *Lactobacillaceae, Clostridiaceae, Peptostreptococcaceae*, and *Veillonellaceae* whereas the abundance of *Oscillospiraceae* was increased in the fecal samples of health subjects.

Despite the association of *Bifidobacterium* and *Lactobacillus* with health [59, 60], increased abundance of these genera is associated with ulcerative colitis and Crohn's disease [61]. Moreover, the increased abundance of *Lactobacillus* has been reported in the fecal samples of subjects with rheumatoid arthritis [17, 62, 63], type 2 diabetes in pregnancy [64], and low fiber diet [65]. Other organisms more abundant in the fecal samples of the Periodontitis group than in H, such as *Megasphaera* was previously associated with the dysbiotic gut microbiome, in pancreatic cancer [66] and Type 2 diabetes mellitus [67], whereas *Acidaminococcus* sp. was associated with Type 2 diabetes mellitus [68].

The reasons for dysbiosis at the oral and gut mucosae in patients with periodontitis are still not clear. They may comprise host susceptibility, such as seen in rheumatoid arthritis [17], or maybe due to the translocation of oral organisms to the gut, leading, under certain circumstances, to gut dysbiosis and contributing to systemic inflammation [69, 70].

Oral and stool communities are especially diverse [71], as shown by the distinct core microbiomes of the oral cavity and feces, and correlation analysis did not lead to the detection of an oral organism where the abundance was directly correlated with the microbial shift in the gut. However, oral pathobionts may still elicit an immune response in animal models, leading to other diseases [72].

We have earlier shown that these patients with periodontitis had higher salivary levels of interleukin 6 (IL-6) and IL-1β, and elevated pro-inflammatory: anti-inflammatory ratio compared to H [27]. The present analyses indicated that the subgingival microbiome correlated with the salivary levels of certain mediators in patients with periodontitis (Figure 4) and in healthy subjects (Supplementary Figure 4), an observation that should contribute to the understanding of the role of specific members of the microbial community and the disease. We have chosen to correlate the inflammatory mediator levels in saliva with microbiome data of a pool of subgingival sites, as recently performed [73] but differing from other studies [28, 74].

Our strategy was based on the fact that mediators in saliva differing between periodontitis and healthy subjects should be produced in the periodontal pockets, triggered by the subgingival microbiome, but differences in the single sites were minimized by evaluating a pool of sites with similar periodontal conditions. Furthermore, the correlation analysis was performed separately to the periodontitis groups and health groups, since several species and mediators were not detected in one of the two groups.

In the context of infection, several chemokines are induced to recruit innate immune cells aiming to kill pathogens, prevent microbial dissemination, drive inflammation, and help repair damage [75]. However, periodontitis is featured by a typical inflammatory imbalance induced by the pathobionts, with increased levels of pro-inflammatory mediators in a Th1 cell response [76].

The integration of microbiome data of gingival bleeding periodontitis with inflammatory mediator levels indicated that the abundance of *Parvimonas micra, Selenomonas sputigena, F. nucleatum* subsp. *vincentii, Fretibacterium fastidiosum, Tannerella forsythia, and Treponema maltophilum* and less studied organisms, such as *Oribacterium* HMT 078 (a Firmicutes of the family *Lachnospiraceae*), and *Anaerolineae* HMT 439 (a member of the Chloroflexi phylum) positively correlated with the salivary levels of several cytokines and chemokines in periodontitis subjects, although each organism yielded a unique correlation profile. On the other hand, the abundance of the recognized pathogen, *P. gingivalis* did not correlate with the levels of any studied mediator. These results should be expected since pathogens, such as *P. gingivalis* and *Treponema denticola* stimulated low levels or even inhibited the expression of inflammatory cytokines and chemokines in *in vitro* models, and their proteases degraded these factors, whereas *F. nucleatum* subsp. vincentii and other species considered less pathogenic induced high expression of inflammatory mediators by gingival fibroblasts [77].

The saliva of the studied patients with periodontitis yielded higher levels of IL-6 than that of health controls [27]. The IL-6 is associated with chronic inflammation [78], and is considered to be a biomarker for chronic periodontitis [26]. The integrated data showed that salivary levels of IL-6 positively correlated with the RA of *P. micra, Selenomonas sputigena, F. nucleatum vincentii, Fretibacterium fastidiosum, Tannerella forsythia, Treponema maltophilum*, and *Oribacterium* HMT 078. These correlations are in accordance with *in vitro* data which indicated that IL-6 is produced by different host cells after challenge with whole bacteria or their components using *P. micra* [79], *F. nucleatum* [80, 81], *T. forsythia* [82], or *T. maltophilum* [83].

*Tannerella forsythia* and *F. nucleatum* subsp. *vincentii* present a synergic relation with biofilm formation [84], and these two species yielded a similar correlation pattern since their abundance was positively correlated with the salivary levels of IL-6 and IL-10, CCL27, and CXCL13. As mentioned, not only IL-6 levels but also CCL27 and CXCL13 high levels were previously associated with periodontitis [27, 85].

The abundance of other organisms, such as *Anaerolineae* HMT 439, *Oribacterium* HMT 078, *P. micra*, and *S. sputigena* positively correlated with the levels of the cytokines, IL-2 and IL-10; and chemokines, CCL7, CCL11, CXCL12, and CXCL16. In the context of the pathogenesis of periodontitis, these chemokines should contribute to the inflammatory process. Indeed, high levels of CCL11, also named Eotaxin-1/C-C motif chemokine 11 [86, 87], CXCL12, also referred to as stromal cell-derived factor-1 (SDF-1) [88] and CCL7, also known as monocyte chemotactic protein-3 (MCP-3) [89] were suggested as biomarkers for periodontitis. The CXCL12 promotes chemotaxis of T lymphocytes and monocytes, whereas CCL7 recruits monocytes [90], and CXCL16 controls the attraction and migration of activated T cells to the inflamed periodontal tissues [91].

*Tannerella forsythia, F. nucleatum, S. sputigena, P. micra, F. fastidiosum, and T. maltophilum* are recognized as candidate pathogens in human periodontitis [92], indicating the potential of the integrative approach to distinguish species within the bacterial community involved in the disease process. Others, such as Oribacterium HMT 078 and Anaerolineae HMT439 are still uncultivated, and little is known about their roles in periodontitis.

Regarding organisms associated with health, the abundance of *R. aeria* and *H. parainfluenzae* positively correlated with (MCP-1) CCL2 levels, whereas the abundance of *H*. *parainfluenzae* was negatively correlated with the levels of CCL13 and CCL24, CXCL11 and CXCL13 (Figure 4). Chemokines whose levels were negatively correlated with the abundance of *H. parainfluenza* may also contribute to periodontal destruction. The CCL24 induces M1 macrophage chemotaxis [93]. The CCL13 (also called Monocyte Chemoattractant Protein 4- MCP4) is involved in the inflammatory process of several diseases [94] and its levels are increased in the gingival crevicular fluid (GCF) of patients with periodontitis [95] whereas CXCL11 is related with Th1 cell accumulation in inflamed mucosa [96].

The correlation of CCL2 (MCP-1) salivary levels with the abundance of beneficial oral bacteria corroborate with other data indicating its protective role. Locally delivered CCL2 prevented alveolar bone loss in a periodontitis mice model due to its ability to decrease macrophage M1:M2 ratio in the gingival tissues, leading to the resolution of inflammation [97]. Furthermore, our group reported that salivary levels of CCL2 were diminished in aggressive periodontitis of the incisor-molar phenotype [27]. However, other data reported that CCL2 levels were increased in the serum of patients with periodontitis [86], indicating that the role of CCL2 in periodontitis should still be addressed.

These data suggest that *R. aeria* and *H. parainfluenzae* are beneficial to the host. On the other hand, a longitudinal study on periodontitis subjects submitted to periodontal treatment reported that the abundance of *Rothia* showed negative associations with *Selenomonas, Fusobacterium*, and *Prevotella* [73]. Thus, it is possible that *Rothia* and/or *H. parainfluenzae* did not directly trigger CCL2 production or inhibit the production of inflammatory mediators but would meet suitable conditions for growth under an environment where inflammation is resolved.

Our data should be interpreted under certain limitations, especially due to the low number of subjects in each group. However, both groups were homogeneous with regard to age, differing from other studies where inflammation may account as a confounding factor [98]. Furthermore, all periodontitis subjects had moderate progressive disease (Grade B), similar disease severity, and the number of affected sites, indicating a similar contribution to salivary mediator levels. Only the abundance of single species was correlated with the levels of inflammatory mediators; therefore, the synergic effect of the microbial community could not be evaluated. Since the production of inflammatory mediators is not the result of signaling by single organisms, other correlations would be possible by combining different organisms, such as those used in certain *in vitro* models [99].

The present study pointed out that dysbiosis does not occur only in periodontal pockets, but the dysbiotic community of biofilms of supragingival and subgingival healthy sites of patients with periodontitis may serve as a reservoir for pathogens. Our data also indicated the dysbiosis of the gut microbiome in periodontitis, similar to other inflammatory diseases. Furthermore, microbial signatures were associated with inflammatory mediators in saliva, evidencing the potential of candidate pathogens [100] and other less-studied organisms, as well the potential benefit promoted by *R. aeria* and *H. parainfluenzae*.

## Data Availability Statement

The datasets presented in this study can be found in online repositories. The names of the repository/repositories and accession number(s) can be found at: https://www.ncbi.nlm.nih.gov/, PRJNA735261.

## Ethics Statement

The studies involving human participants were reviewed and approved by Instituto de Ciências Biomédicas da Universidade de São Paulo. The patients/participants provided their written informed consent to participate in this study.

## Author Contributions

DK contributed to the conception, design, data acquisition, analysis and interpretation, and drafted the manuscript. LS, AH, MF, and PA contributed to data acquisition. RB, RR, and RS contributed to data analysis. MPAM contributed to the conception, design, data acquisition and interpretation, and drafted the manuscript. All authors critically revised the manuscript and gave final approval. The authors agreed to be accountable for all aspects of the study.

## Funding

This study was supported by the Fundação de Amparo à Pesquisa do Estado de São Paulo (FAPESP), Grant 2015/18273-9 and the National Council for Scientific and Technological Development (CNPq) Grant 406704/2016-3. DK and PA were supported by scholarships from FAPESP 2016/13159-6 and 2015/0259-0.

## Conflict of Interest

The authors declare that the research was conducted in the absence of any commercial or financial relationships that could be construed as a potential conflict of interest.

## Publisher's Note

All claims expressed in this article are solely those of the authors and do not necessarily represent those of their affiliated organizations, or those of the publisher, the editors and the reviewers. Any product that may be evaluated in this article, or claim that may be made by its manufacturer, is not guaranteed or endorsed by the publisher.
