# Supplementary figures and images for: Oral Dysbiosis in Severe Forms of Periodontitis Is Associated With Gut Dysbiosis and Correlated With Salivary Inflammatory Mediators: A Preliminary Study
Source: Front Oral Health. 2021 Oct 11;2:722495. doi: 10.3389/froh.2021.722495 (PMC8757873; doi:10.3389/froh.2021.722495)

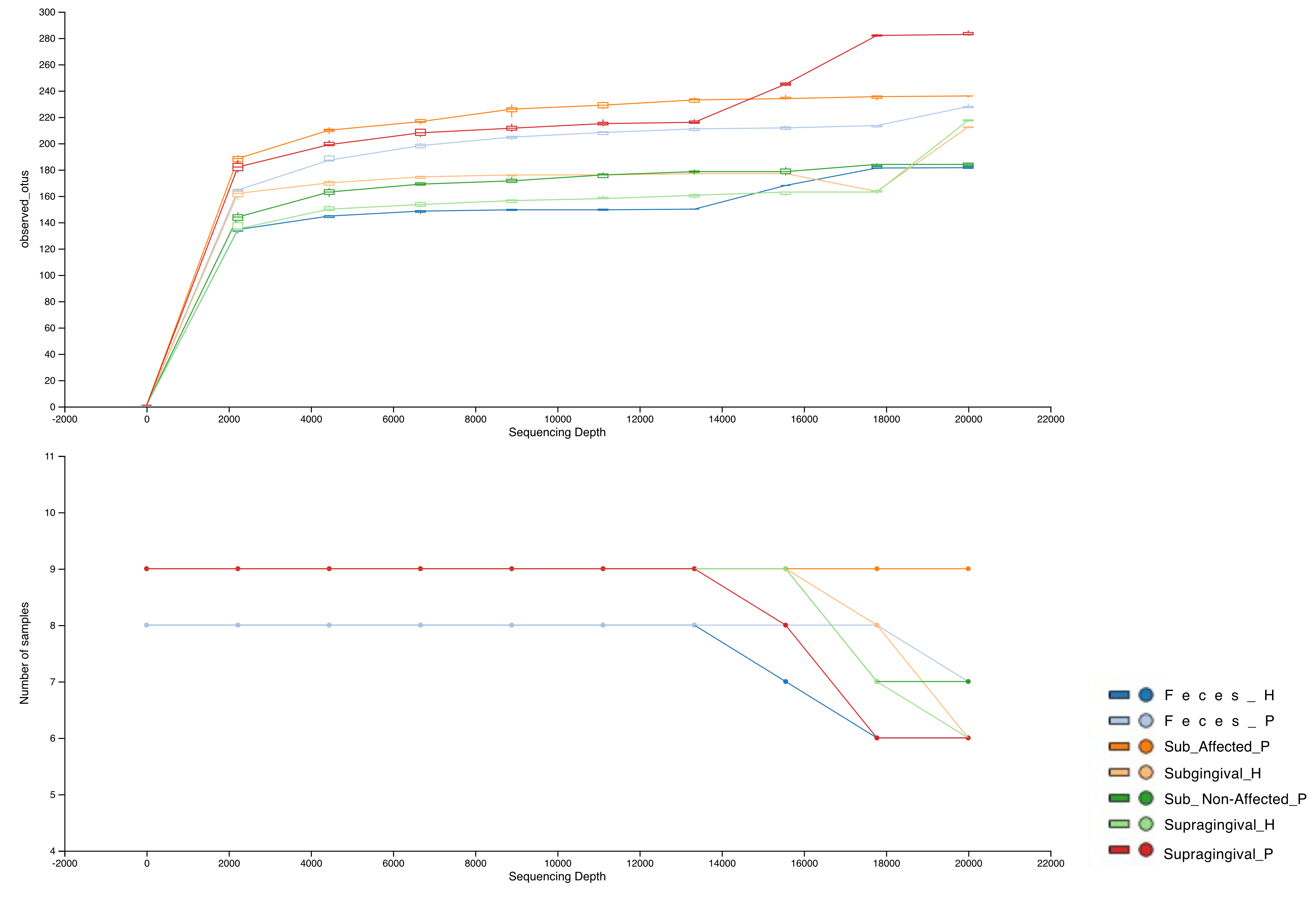

Supplement: Supplementary file 1 [file Image_1.TIFF]

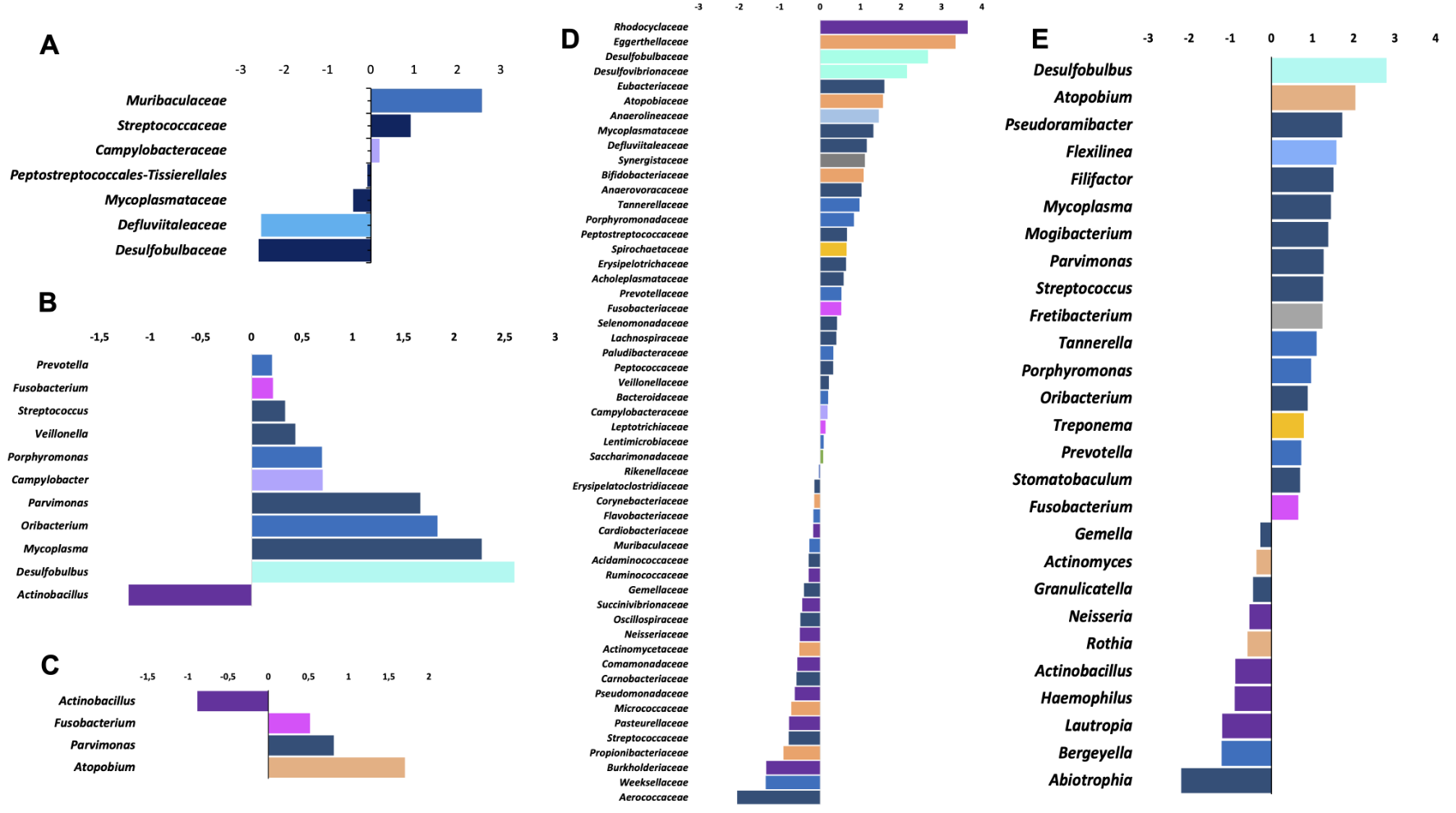

Supplement: Supplementary file 2 [file Image_2.TIFF]

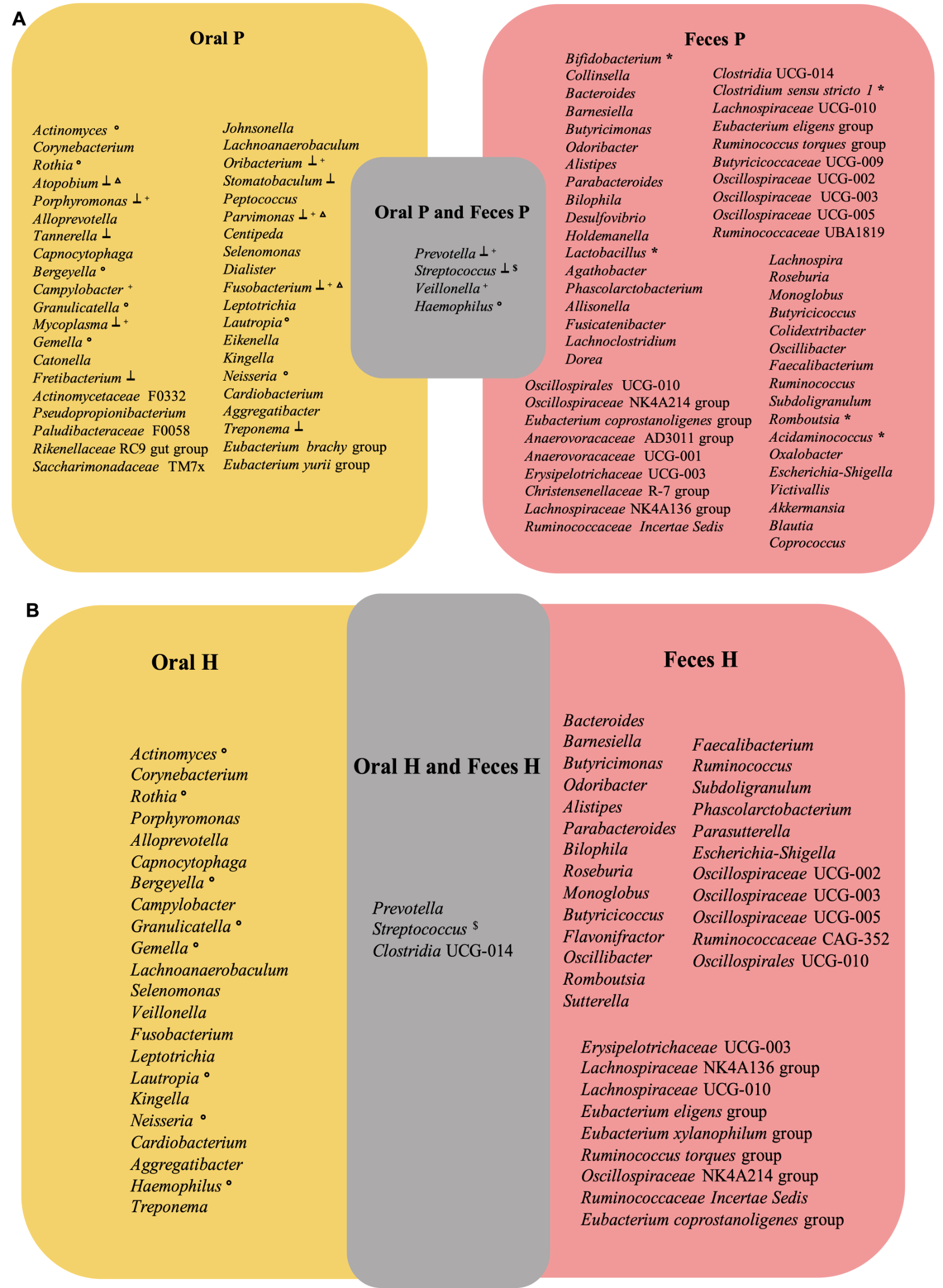

Supplement: Supplementary file 3 [file Image_3.TIFF]

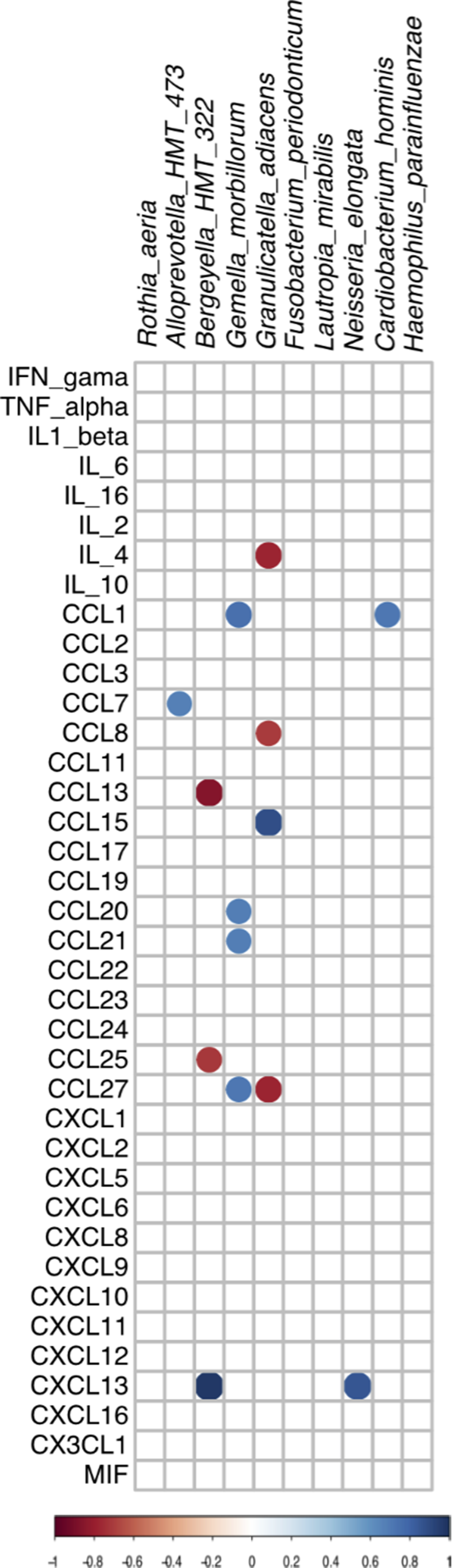

Supplement: Supplementary file 4 [file Image_4.TIFF]
