# Supplementary material for: Oral Dysbiosis in Severe Forms of Periodontitis Is Associated With Gut Dysbiosis and Correlated With Salivary Inflammatory Mediators: A Preliminary Study
Source: Front Oral Health. 2021 Oct 11;2:722495. doi: 10.3389/froh.2021.722495 (PMC8757873; doi:10.3389/froh.2021.722495)
Supplement: Supplementary file 6 [file Table_1.DOCX]

Supplementary Table 1

**Table S1**. Median and interquartile range of Faith and Pielou_Eveness of the groups analysed, with Wilcoxon-Mann Whitney test

| Groups compared | | Faith | | | | Pielou_Eveness | | | |
| --- | --- | --- | --- | --- | --- | --- | --- | --- | --- |
|  |  | Median (interquartile range) | | W | p-value | Median (interquartile range) | | W | p-value |
| Supragingival_H | Supragingival_P | 10.99 (8.66 - 11.61) | 14.05 (9.02 - 18.94) | 34 | 0.60 | 0.68 (0.55 - 0.80) | 0.82 (0.80 - 0.84) | 18 | 0.05 |
| Subgingival_H | Subgingival_non_affected_P | 12.33 (11.58 - 14.68) | 13.64 (12.46 - 14.31) | 77 | 0.85 | 0.75 (0.71 - 0.83) | 0.80 (0.77 - 0.80) | 34 | 0.60 |
| Subgingival_non_affected_P | Subgingival_affected_H | 15.99 (15.33 - 16.68) | 12.33 (11.58 - 14.68) | 23 | 0.13 | 0.77 (0.73 - 0.80) | 0.75 (0.71 - 0.83) | 39 | 0.93 |
| Feces_H | Feces_P | 11.25 (9.53 - 14.70) | 13.10 (11.85 - 14.10) | 27 | 0.64 | 0.78 (0.73 - 0.79) | 0.75 (0.74 - 0.77) | 36 | 0.72 |
