# Supplementary material for: Oral Dysbiosis in Severe Forms of Periodontitis Is Associated With Gut Dysbiosis and Correlated With Salivary Inflammatory Mediators: A Preliminary Study
Source: Front Oral Health. 2021 Oct 11;2:722495. doi: 10.3389/froh.2021.722495 (PMC8757873; doi:10.3389/froh.2021.722495)
Supplement: Supplementary file 7 [file Table_2.DOCX]

Supplementary Table 2

**Table S2**. Mean and standard deviation of levels of cytokines and chemokines in pg/ml in saliva.

| **Cyto/Chemokine** | **Periodontitis** | **Health** |
| --- | --- | --- |
| IFN_γ | 9,05(± 5,02) | 8,03(± 2,03) |
| IL1_β | 718,72(± 856,08) | 187,46(± 192,49) |
| IL_2 | 1,6(± 0,7) | 1,7(± 0,77) |
| IL_4 | 2,54(± 0,54) | 2,59(± 0,27) |
| IL_6 | 10,8(± 10,75) | 2,41(± 2,1) |
| IL_8_CXCL8 | 364,59(± 206,29) | 342,02(± 157,3) |
| IL_10 | 3,14(± 1,11) | 3,18(± 0,7) |
| IL_16 | 566,56(± 615,69) | 179,86(± 171,43) |
| TNF_α | 9,99(± 5,25) | 8,29(± 3,96) |
| I309_CCL1 | 7,98(± 2,07) | 8,58(± 1,11) |
| MCP_1_CCL2 | 142,42(± 159,09) | 162,02(± 146,59) |
| MIP_1α_CCL3 | 2,7(± 1,76) | 1,34(± 0,73) |
| MCP_3_CCL7 | 20,81(± 17,27) | 26,16(± 30,74) |
| MCP_2_CCL8 | 1,01(± 0,43) | 0,73(± 0,17) |
| EOTAXIN_CCL11 | 4,1(± 1,42) | 3,73(± 0,7) |
| MCP_4_CCL13 | 10,8(± 5,34) | 13,69(± 3,77) |
| MIP_1σ_CCL15 | 11,44(± 9,76) | 9,08(± 6,52) |
| TARC_CCL17 | 3,6(± 1,76) | 3,7(± 1,09) |
| MIP_3β_CCL19 | 6,07(± 5,04) | 4,32(± 0,69) |
| MIP_3α_CCL20 | 12,93(± 14,95) | 5,77(± 2,8) |
| 6CKINE_CCL21 | 123,47(± 141,41) | 82,53(± 47,94) |
| MDC_CCL22 | 7,3(± 4,85) | 4,37(± 1,03) |
| MPIF1_CCL23 | 4,87(± 1,99) | 5,04(± 1,91) |
| EOTAXIN2_CCL24 | 9,01(± 3,44) | 8,19(± 2,69) |
| TECK_CCL25 | 49,16(± 16,55) | 63,34(± 16,02) |
| CTACK_CCL27 | 1,95(± 0,9) | 1,55(± 0,25) |
| GRO_α_CXCL1 | 1011,31(± 537,19) | 1071,21(± 767,04) |
| GRO_β_CXCL2 | 162,53(± 129,96) | 110,41(± 32,2) |
| ENA_78_CXCL5 | 5792,61(± 4911,82) | 5482,95(± 3497,8) |
| GCP2_CXCL6 | 69,04(± 63,09) | 28,15(± 16,12) |
| MIG_CXCL9 | 159,69(± 142,11) | 154,23(± 146,88) |
| IP_10_CXCL10 | 144,86(± 208,99) | 51,84(± 31,94) |
| ITAC_CXCL11 | 2,44(± 2,52) | 1,96(± 1,99) |
| SDF1α+β_CXCL12 | 18,44(± 4,33) | 18,7(± 4,9) |
| BCA_1_CXCL13 | 2,62(± 2,51) | 2,2(± 3,43) |
| SCYB16_CXCL16 | 20,69(± 15,01) | 21,64(± 12,01) |
| FRACTALKINE_CX3CL1 | 1338,39(± 784,49) | 1220,19(± 802,35) |
| MIF | 10822,22(± 17984,41) | 10164,86(± 7797,81) |
